# Dislocation loop and irradiation-induced synergistic-competitive mechanism in Cu-rich precipitates: a phase-field study

**DOI:** 10.1038/s41598-024-63632-5

**Published:** 2024-06-04

**Authors:** Wenkui Yang, Qingwei Guo, Kaile Wang, Pengya Lei, Hua Hou, Yuhong Zhao

**Affiliations:** 1 School of Materials Science and Engineering, Collaborative Innovation Center of Ministry of Education and Shanxi Province for High-Performance Al/Mg Alloy Materials, North University of China, Taiyuan, 030051 People’s Republic of China; 2 Beijing Advanced Innovation Center for Materials Genome Engineering, University of Science and Technology Beijing, Beijing, 100083 People’s Republic of China; 3 A School of Materials Science and Engineering, Taiyuan University of Science and Technology, Taiyuan, 030024 People’s Republic of China; 4 Institute of Materials Intelligent Technology, Liaoning Academy of Materials, Shenyang, 110004 People’s Republic of China

**Keywords:** Phase-field model, Vacancy-interstitial atoms, Cascade mixing, Dislocation loop, Cu-rich precipitates, Materials science, Statistical physics, thermodynamics and nonlinear dynamics

## Abstract

Both irradiation and dislocations have been proposed as routes to rationally manipulate spatial distribution and micromorphology of precipitate. An interesting effect emerges in Fe–10at.%Cu–3at.%Mn–1.5at.%Ni–1.5at.%Al alloy due to the synergistic-competitive roles of dislocation loop and irradiation. Base on cascade mixing, vacancy-interstitial atoms and dislocation stress field model, we examine nucleation and growth dynamics of Cu-rich precipitates, where both dislocation loop and irradiation act in conjunction. Analytical treatments identify regimes, where the distribution of elements and point defects due to irradiation and dislocations are specific to the Cu-rich precipitates. Simulation results reveal that density, size and distribution of Cu-rich precipitates are a manifestation of the competing effects of the dislocation loop and the irradiation rate. More specifically, the dislocation loop preferentially assists the formation of precipitates and new dislocations at lower irradiation rates. Only the irradiation induces the formation of Cu-rich precipitates with the irradiation rate continues to increase. Equipped with molecular dynamics, where reproduces major interaction features of the solutes with point defects under displacement cascade, can verify multi-component morphologies of Cu-rich precipitates. This modeling framework provides an avenue to explore the role of dislocation loop and irradiation on the microstructural evolution of Cu-rich precipitates.

## Introduction

The stability and irradiation tolerance of Fe–Cu based alloy is a critical factor in determining the lifespan and safety of nuclear reactor pressure vessels (RPVs)^1–3^. Precipitation hardening and embrittlement, enhanced or induced by in-reactor neutron irradiation, is a major concern. There is a significant body of research on irradiation-enhanced precipitation that includes irradiation rate and diffusivity of vacancy-associated migration energy studies^4–6^. Some attempts have been made to study the interaction of irradiation with precipitation^7–9^. Both of the aforementioned avenues of irradiation-enhanced precipitation are highly intertwined and act in conjunction; therefore, detangling the role of each on the observed precipitation hardening and embrittlement is of interest from a scientific and application standpoint^10–12^.

However, multi-component alloys are designed to achieve the best combination of mechanical properties, thermal stability, corrosion resistance, etc. Since Cu of RPVs is highly insoluble and diffuses rapidly in the Fe matrix, the initial precipitates are copper-rich, often alloyed with Mn, Ni, and Al^13–17^. Meanwhile, in addition to enriching the Cu precipitates, solutes such as Mn, Ni, and Al could form a core-shell structure due to reduction of the high interfacial energy between Fe and Cu. Such core-shell structures were widely reported in experiments^18–20^, first principles^21–24^, molecular dynamics and phase-field^25–34^, both under irradiation and thermal aging conditions.

Dislocations, as sources or sinks for the formation or dissolution of precipitates, can contribute to faster kinetics and the formation of ordered microstructures^35,36^. Meanwhile, dislocations can facilitate the movement of atoms within the material, allowing for faster diffusion and rearrangement of atoms^36–38^. This enhanced atomic mobility leads to accelerated transformation processes, such as phase separation or solid-state phase transition. The presence of local high-density dislocations, dislocation loops, and interactions between dislocations and precipitates can perturb the local stress distribution, resulting in changes to the growth kinetics and altering the shape and preferred orientation of precipitates^39–45^. Thus, it is important to understand the underlying mechanisms of dislocation to optimize alloy designs.

Although there have been studies on the precipitation process of multi-component Fe–Cu-based alloys, irradiation-enhanced diffusion, and dislocation-assisted phase separation, the distribution of CRPs and vacancy-interstitial atoms in multi-component alloys under the synergistic competition between dislocation loops and irradiation has not been reported. Based on this, the phase-field model is established by coupling cascade mixing, vacancy-gap atoms and dislocation elastic field in this paper. The simulation model and results in this work were integrated into the EasyPhase software package, which was developed by the group of Prof. Yuhong Zhao^46^. The aim is to study the irradiation-induced precipitation, irradiation and dislocation loop synergistic induced precipitation, and irradiation and dislocation loop competitive induced precipitation. And molecular dynamics simulations are combined to validate the phase-field simulation results. Meanwhile, the simulation results are combined with the strengthening and hardening model to achieve a closed-loop of process → microstructure → performance.

## Methods

### Phase-field model

The time-evolution of the conserved order parameter ${c}_{i}\left({\varvec{r}},t\right)$, ${c}_{V}\left({\varvec{r}},t\right)$ and ${c}_{I}\left({\varvec{r}},t\right)$ are determined by the Cahn–Hilliard-type equation with a forced mixing term^28,32–34,47^:

1 $$\begin{aligned} \frac{{\partial c_{i} \left( {{\varvec{r}},t} \right)}}{\partial t} = & \nabla \cdot M \cdot \nabla \frac{\delta F}{{\delta c_{i} ({\varvec{r}},t)}} + \frac{{\partial c_{i} \left( {{\varvec{r}},t} \right)}}{\partial t}|_{mix} \\ \frac{{\partial c_{V} \left( {{\varvec{r}},t} \right)}}{\partial t} = & \nabla \cdot \left( {M_{V} \cdot \nabla \frac{\delta F}{{\delta c_{V} ({\varvec{r}},t)}}} \right) + P_{V} + R_{VI} - S_{V} \\ \frac{{\partial c_{I} \left( {{\varvec{r}},t} \right)}}{\partial t} = & \nabla \cdot \left( {M_{I} \cdot \nabla \frac{\delta F}{{\delta c_{I} ({\varvec{r}},t)}}} \right) + P_{I} + R_{VI} - S_{I} \\ \end{aligned}$$

where ${c}_{i}\left({\varvec{r}},t\right)$, ${c}_{V}$ and ${c}_{I}$ are the instantaneous concentration of $i$ element, vacancy, and interstitial atoms. ${P}_{V}={P}_{I}=\varepsilon {K}_{0}$ ($\varepsilon$ is cascade efficiency and ${K}_{0}$ is irradiation rate) are the production rates of vacancies-interstitial atoms. ${R}_{VI}=\frac{4\pi {r}_{VI}\left({D}_{V}+{D}_{I}\right)}{V}{c}_{V}{c}_{I}$ is the recombination rate of the vacancies- interstitial atoms. ${S}_{V}$ and ${S}_{I}$ are the sink rate of the vacancies and interstitial atoms. Total mobility $M$ including bulk mobility ${M}_{ij}^{\text{th}}$ (See Appendix A of supplementary information), irradiation mobility $M_{ij}^{{{\text{irr}}}}$, and dislocation pipe mobility $M^{dis}$^48^.

2 $$\begin{array}{*{20}c} {M = M_{ij}^{{{\text{th}}}} + M_{ij}^{{{\text{irr}}}} + M^{dis} } \\ \end{array}$$

The total freedom of the system consists of the chemical energy density $f_{ch} \left( {c_{i} ,c_{V} ,c_{I} ,T} \right)$, the interfacial energy density $f_{in} \left( {c_{i} ,c_{V} ,c_{I} } \right)$ (See Appendix A of supplementary information), and the elastic energy density $f_{el} \left( {c_{i} ,c_{V} ,c_{I} } \right)$^49^:

3 $$\begin{array}{*{20}c} {F\left( {c_{i} ,c_{V} ,c_{I} ,T} \right) = \mathop \smallint \limits_{V} \left( {f_{ch} \left( {c_{i} ,c_{V} ,c_{I} ,T} \right) + f_{in} \left( {c_{i} ,c_{V} ,c_{I} } \right) + f_{el} \left( {c_{i} ,c_{V} ,c_{I} } \right)} \right)dV} \\ \end{array}$$

### Radiation and dislocation enhanced diffusion (RED)

We utilize the Radiation-Enhanced Diffusion (RED) model to calculate the vacancy concentration (${c}_{V}$) during irradiation, which takes into account the thermal diffusion coefficients proportional to the vacancy supersaturation. When the production of defects is balanced by their annihilation at sinks and recombination in the matrix, especially at vacancies trapped by solute atoms, the RED model incorporates not only the annihilation of vacancies at sinks and recombination in the ordinary matrix, but also accounts for the influence of solute vacancy trapping, which is dependent on the displacement per atom (dpa) rate. This trapping enhances the recombination of Vacancy-interstitial atoms, leading to a reduction in the vacancy concentration (${c}_{V}$). The irradiated mobility $M_{ij}^{{{\text{irr}}}}$ can be expressed as^7,8^

4 $$\begin{array}{*{20}c} {M^{{{\text{irr}}}} = \frac{{D_{i}^{irr} }}{RT} = \frac{{D_{i}^{{{\text{th}}}} }}{RT}\frac{{C_{{\text{v}}}^{{{\text{irr}}}} }}{{C_{{\text{v}}}^{{{\text{th}}}} }}} \\ \end{array}$$

where $D_{i}^{irr}$ and $D_{i}^{{{\text{th}}}}$ are the irradiated and thermal diffusivity, respectively. $\frac{{C_{{\text{v}}}^{{{\text{irr}}}} }}{{C_{{\text{v}}}^{{{\text{th}}}} }}$ is the radiation-enhanced factor.

We define the mobility $M^{dis}$ in the simulation domain using the order parameters as follows^42^:

5 $$\begin{array}{*{20}c} {M^{dis} = M_{D} \left\{ {max\left( {\left| {\eta \left( {1 - \eta } \right)} \right|} \right)} \right\}} \\ \end{array}$$

where $\eta$ is the order parameter that describe dislocations slip in (001) slip plane. $M_{D}$ is the enhanced mobility along the dislocation core. The mobility of atoms given by Eq. (5) will be highest at points where $\eta = 0.5$.

### Dislocation model

The elastic energy density the system, consists of, in addition to the elastic component, the dislocation core energy components:

6 $$\begin{array}{*{20}c} {f_{el} = \frac{1}{2}C_{ijkl} \left( {\varvec{r}} \right)\varepsilon_{ij}^{el} \left( {\varvec{r}} \right)\varepsilon_{kl}^{el} \left( {\varvec{r}} \right) + \mathop \sum \limits_{\alpha = 1}^{N} B{\text{sin}}^{2} \left( {\pi \eta_{\alpha } } \right)} \\ \end{array}$$

where $B$ is the core energy coefficient of dislocations slip (Value range: 0.31–2.52). According to Khachaturyan’s^50^ micromechanical theory, the elastic strain $\sigma_{ij}^{el} \left( {\varvec{r}} \right)$ can be expressed as:

7 $$\begin{array}{*{20}c} {\varepsilon_{ij}^{el} = \delta \varepsilon_{ij} - \varepsilon_{ij}^{0} - \varepsilon_{ij}^{d} = \delta \varepsilon_{ij} - \varepsilon_{0}^{i} \delta_{ij} \left( {c_{i} - c_{i}^{0} } \right) - \frac{{\left( {b_{i} n_{j} + b_{j} n_{i} } \right)}}{2d}} \\ \end{array}$$

where $\delta \varepsilon_{ij}$ is inhomogeneous strain, $\varepsilon_{ij}^{0}$ is eigenstrain due to the solute misft, and $\varepsilon_{ij}^{d}$ is dislocation strain. $\varepsilon_{0}^{i}$ is the magnitude of the eigenstrain, $\delta_{ij}$ is Kronecker delta function. $c_{i}^{0}$ is the initial component value of element $i$. $d$ is the distance between the planes of the sliding plane.

### Cascade mixing

Enrique and Bellon characterized the local composition change caused by cascade mixing as^51^:

8 $$\begin{array}{c}\frac{\partial {c}_{i}}{\partial t}{|}_{mix}=-\Gamma \left({c}_{i}-{\langle {c}_{i}\rangle }_{R}\right)\end{array}$$

where $\Gamma$ is the relocation frequency that is related to the dpa rate ($\phi dpa$) by $e\phi dpa$. $e$ is a parameter characterizing the number of replacement per atomic displacement. We assume that $e=50$ for neutron irradiations in the simulation ${\langle {c}_{i}\rangle }_{R}$ is nonlocal average of the concentration weighted by a normalized function.

9 $$c_{iR} = \int {\omega_{R} \left( {{\varvec{r}} - {\varvec{r}}^\prime } \right)c_{i} \left( {{\varvec{r}}^\prime } \right)d{\varvec{r}}^\prime }$$

where ${\omega }_{R}$ is the normalized function characterizing atomic relocation^52^.

10 $$\begin{array}{*{20}c} {\omega_{R} \left( {{\varvec{r}} - \varvec{r^{\prime}}} \right) = \left( {\frac{3}{{2\pi R\prime^{2} }}} \right)^{3/2} exp\left( { - \frac{{3\left| {{\varvec{r}} - \varvec{r^{\prime}}} \right|^{2} }}{{2R^{2} }}} \right)} \\ \end{array}$$

where r and $R^\prime$ are the spatial position and average jumping distance, respectively. In the simulation we use the nearest neighbor distance of the bcc-a lattice ($2.49 \times 10^{ - 10} \;{\text{m}}$) as $R^\prime$.

### Numerical implementation

**Figure 1:**
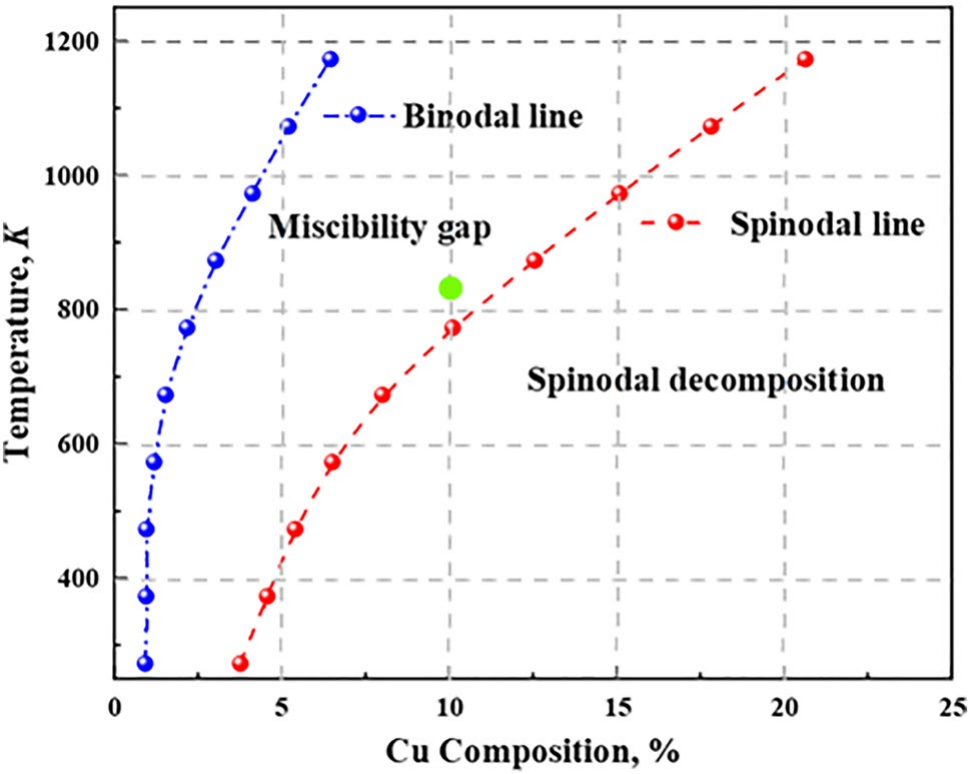
Phase diagram of Fe–Cu–Mn–Ni–Al alloy including the miscibility gap (Spinodal line) and the spinodal decomposition (Binodal line).

We also note that in dimensional terms, a $128\times 128\times 32$ grid corresponds to a system of size $128\times 128\times 32{\text{nm}}^{3}$. The phase diagram of Fe–Cu–Mn–Ni–Al alloy is obtained as shown in Fig. 1, showing the miscibility gap (Spinodal line) and the spinodal decomposition (Binodal line). The green ball is the simulation point selected in this paper: 10 at.% Cu, temperature: 823 K.

## Results

**Figure 2:**
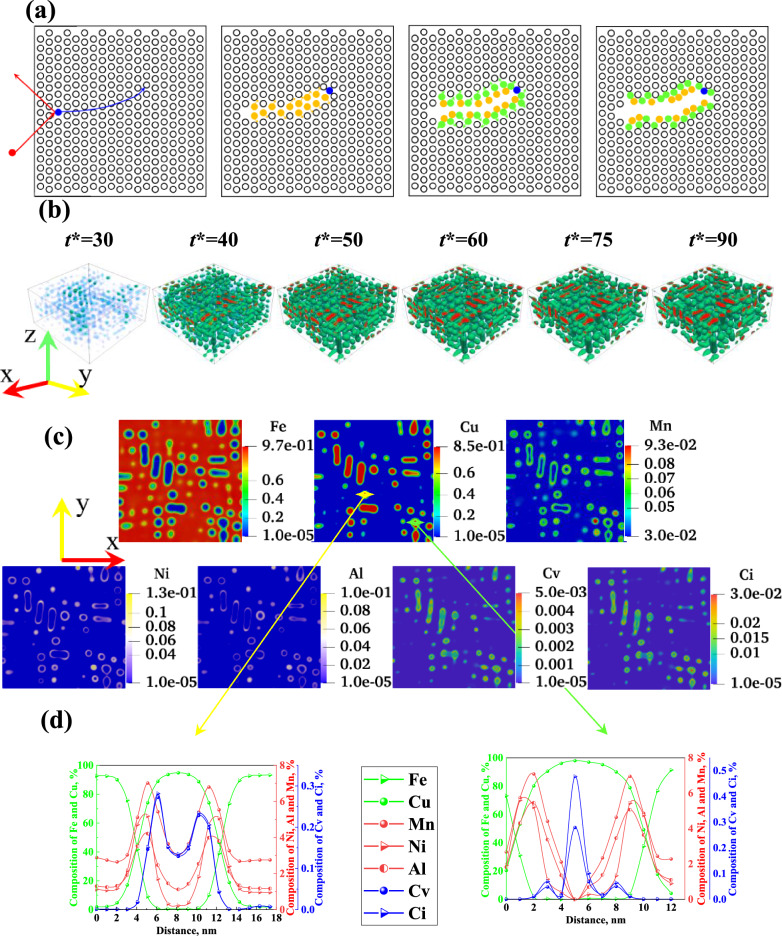
Precipitation of CRPs under neutron-irradiated (${10}^{-7}\text{ dpa}/\text{s}$): (**a**) Schematic diagram of the atomic displacement cascade under neutron irradiation^28^. (**b**) 3D simulation results. (**c**) Distribution of elements in the (001) plane at t* = 90. (**d**) Composition curves at the positions of yellow and green arrows.

**Figure 3:**
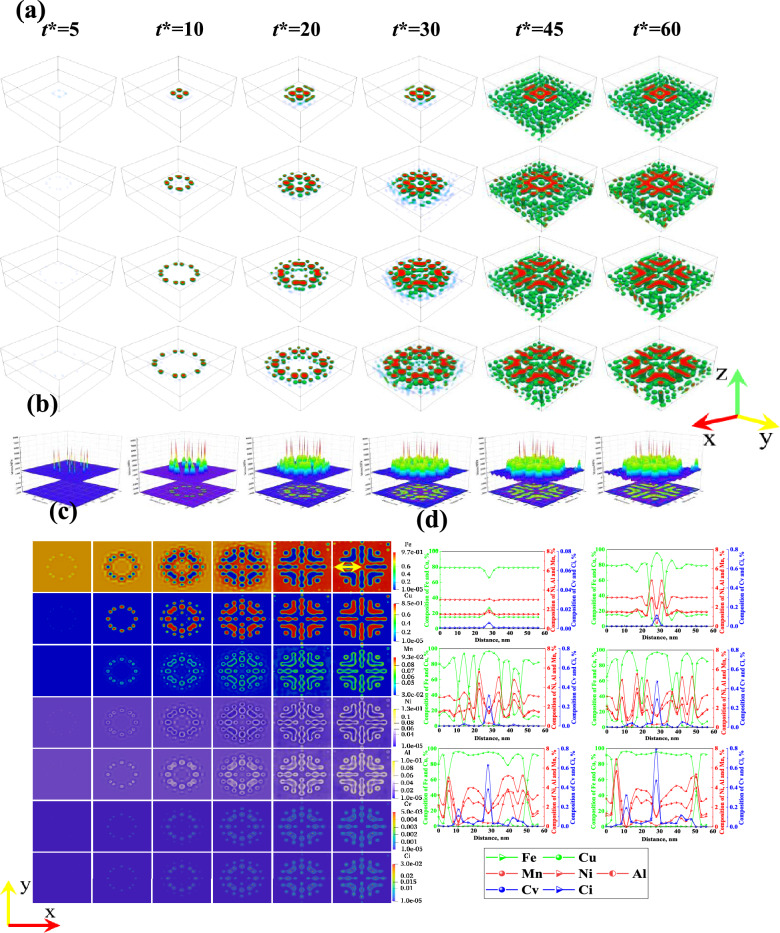
Precipitation of CRPs under the synergistic effect of different dislocation loop (r = 10, 20, 30 and 40 nm,) and neutron irradiation (${10}^{-7}\text{ dpa}/\text{s}$): (**a**) 3D simulation results. (**b**) Evolution of the stress field in the (001) plane with time (loop radius r = 30 nm). (**c**) Evolution of the element distribution in the (001) plane with time (loop radius r = 30 nm). (**d**) Evolution of the compositional curves with time at the yellow arrows in (**c**).

Neutrons (red spheres) colliding with lattice atoms (black hollow spheres) to generate primary colliding atoms (yellow spheres), which further collide with lattice atoms to produce secondary collision atoms (green spheres). The dynamic migration and collision of atoms coalesce to give rise to multi-level colliding atoms, leading to the creation of regions characterized by varying atom densities—atom-rich regions and atom-poor regions, as depicted in Fig. 2a. Eventually, atom-rich and atom-poor regions are formed. The green contour in Fig. 2b is the composition with ${c}_{Cu}=0.5$, indicating the interface between the CRPs and the substrate, while the red spheres are CRPs. The findings from the 3D analysis reveal that solute atoms cluster within the atom-rich region induced by irradiation, eventually forming CRPs. Simulations of the (001) surface in Fig. 2c demonstrate that Cu atoms are centrally concentrated within the precipitates, surrounded by Mn, Ni, Al, and vacancy-interstitial atoms which cluster around CRPs. This arrangement ultimately results in the formation of a double core-shell structure composed of Cu in the inner shell and a combination of vacancy-interstitial atoms and Mn, Ni, Al in the outer shell. The presence of a double core-shell structure is confirmed by the compositional curves of Fig. 2d, which provide evidence for its existence. Furthermore, the aggregation of vacancy-interstitial atoms within the core of CRPs can be observed (for detailed explanations, please refer to Fig. 3).

3D simulation results of Fig. 3a show that solute atoms preferentially cluster around the dislocation loop at the early stage of aging, forming a "solute atmosphere", which hinders dislocation motion. Subsequently, discontinuous spherical CRPs are formed along the dislocation loop, while new dislocations are generated around the CRPs, which induces the formation of new precipitates. This is the same as the previous findings^40^. Atom-rich regions gradually form granular precipitates under irradiation, which absorb solute atoms and grow into spherical precipitates and rod-like precipitates. Different radii of dislocation rings contribute to the formation of distinct microscopic morphologies: for a radius of 10 nm, it results in square hollow circle precipitates; for a radius of 10 nm, it leads to ring-shaped precipitates; while for radii of 20 nm and 30 nm, they result in rod-shaped and “V” shaped precipitates. The results of the 3D atomic distribution see Figs. S2–S5 of Appendix C. Figure 3b and c illustrate the temporal variations of stress fields and elemental distribution on the (001) plane (f The elemental distributions of the other dislocation loops are shown in Fig. S6–S8 in Appendix C). Results indicate that Cu, Mn, Ni, Al, and vacancy-interstitial atoms preferentially cluster around the dislocation loops, forming CPRs under the influence of the dislocation stress field. Subsequently, Mn, Ni, Al, and vacancy-interstitial atoms further accumulate around the CPRs, forming a double-shell structure. Vacancy-interstitial atoms continue to accumulate at the center of the CPRs, leading to a sustained increase in stress. The composition curve in Fig. 3d confirms this phenomenon, and the component fluctuations around the spherical CRPs indicate the formation of new dislocations. In Fig. 3b, red represents the stress concentration area and blue represents the low stress area. After the formation of CRPs around the dislocation loop (t* = 20), a stress concentration zone appears around the precipitates. This proves the generation of dislocations around precipitates. The distribution of vacancy-interstitial atoms is closely related to the dislocation (i.e., vacancy-interstitial atoms cluster at the dislocation, or vacancy-interstitial atoms cluster at the interface between the precipitate and the matrix to form a vacancy-interstitial shell). The concentration of vacancy-interstitial atoms increases with aging time at the dislocations. From Fig. 3d, it can be seen that the concentration of vacancy-interstitial atoms at the initial dislocation loop is around 0.8%, while at the new dislocation site, the concentration of vacancy-interstitial atoms is around 0.4% at equilibrium. This demonstrates an increase in vacancy-interstitial atom concentration with the extension of time at the dislocation.

**Figure 4:**
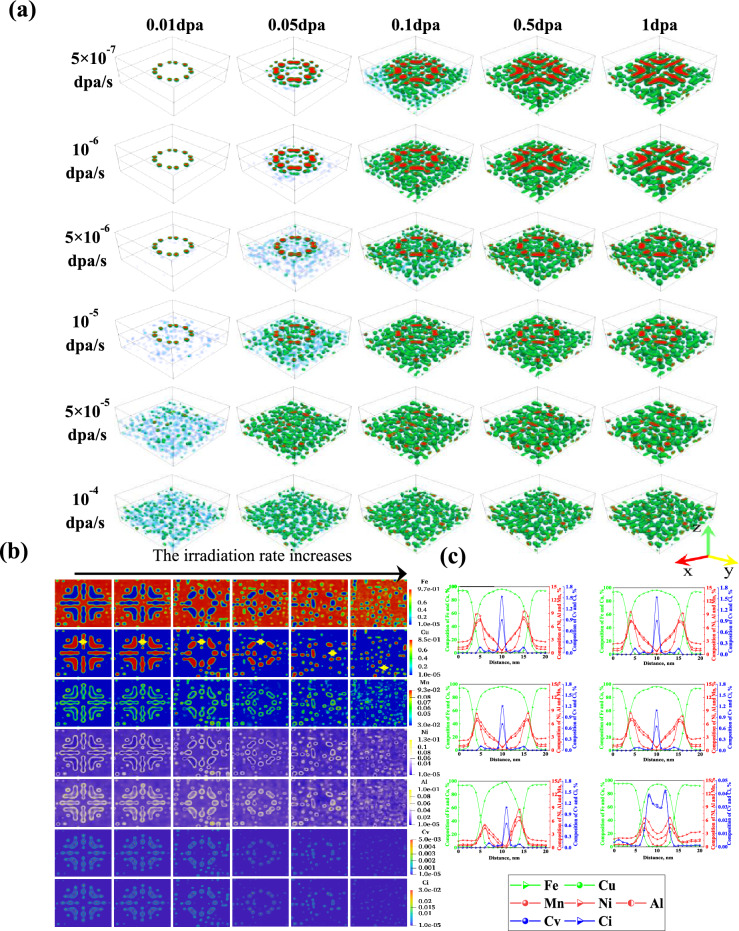
The competition mechanism of CRPs under the action of dislocation loop (r = 30 nm) and different irradiation rates: (**a**) 3D simulation results. (**b**) The atomic distribution diagram of the (001) plane (dislocation loop plane). (**c**) Evolution of the compositional curves with time at the yellow arrows in Fig. 3b.

3D results in Fig. 4a demonstrate that the evolution of CRPs under irradiation rates ranging from 5 × 10^−7^ to 10^−6^ dpa/s is basically the same as the results under irradiation rates of 10^−7^ dpa/s in Fig. 3a. Dislocation loops preferentially assist the formation and growth of CRPs under irradiation rates ranging from $5\times {10}^{-6}$ to ${10}^{-5}\text{ dpa}/\text{s}$, but there was no generation of new dislocations around the CRPs. Only irradiation induced the formation and growth of CRPs under irradiation rates ranging from $5\times {10}^{-5}\text{ dpa}/\text{s}$ to ${10}^{-4}\text{ dpa}/\text{s}$. The atomic distribution diagram of the (001) plane and composition curves in Fig. 4b and c indicate that the concentration of vacancy-interstitial atoms on the dislocation loop increases with the increase of irradiation rate ($<{10}^{-6}\text{ dpa}/\text{s}$), while concentration of vacancy-interstitial atoms at the dislocation loop decreases with increasing irradiation rate ($<{5\times 10}^{-5}\text{ dpa}/\text{s}$). Vacancy-interstitial atoms cluster at the interface of CRPs at high irradiation rate (${10}^{-4}\text{ dpa}/\text{s}$), while higher concentrations of vacancy-interstitial atoms are also present in the interior of CRPs.

**Figure 5:**
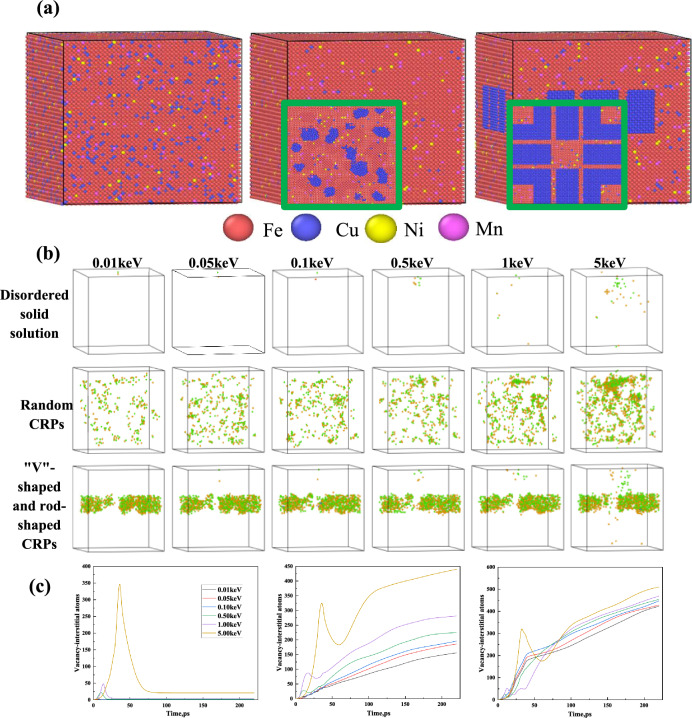
(**a**) Molecular dynamics modeling of precipitated phases containing different shapes of Cu. (**b**) 3D simulation results of vacancy-interstitial atomic distribution and the number of vacancy-interstitial atoms with time at displacement cascades. (**c**) Evolution of vacancy-interstitial atoms in disordered solid solutions, random CRPs, and "V"-shaped and rod-shaped CRPs.

The Large Scale Atomic/Molecular Massively Parallel Simulator (LAMMPS) package is used to perform all molecular dynamics simulations in this work. We use the EAM potential of G. Bonny et al.^53^ to describe the force field between Fe–Cu–Mn–Ni atoms, which can accurately characterize elemental enrichment in Fe–Cu–Mn–Ni alloys as well as cluster formation during irradiation^54^. Three sets of initial results were constructed, including: Disordered solid solution of Fe–10at.%Cu–3at.%Mn–1.5at.%Ni. Randomly distributed Cu-rich precipitates (CRPs). Rod-shaped and "V"-shaped CRPs under the influence of dislocation loops with r = 30 nm as shown in Fig. 5a. Fe atoms with energies of 0.01, 0.05, 0.10, 0.50, 1.00, and 5.00 keV were set at the center of the surface in the Z-direction as the high-energy incident particles. Periodic boundary conditions were applied in all directions to replicate the infinite volume system. A constant temperature zone of 4 Å was created in the outermost layer of the model to maintain a certain temperature in the simulation zone before simulation. The Z direction was chosen as the incidence direction of t the high-energy incident particles. All atoms are in the constant volume and energy (microcanonical system, NVE) system during the displacement cascade. 3D simulation results in Fig. 5b indicate that only one pair of vacancy-interstitial atoms exists in the disordered solid solution at equilibrium. However, the curve in Fig. 5c represents the non-monotonic behavior of vacancy-interstitial atoms. Vacancy-interstitial atoms initial increase followed by a subsequent decrease, and the peak value gradually increasing with displacement cascades, prior to reaching equilibrium. In the presence of CRPs, the number of vacancy-interstitial atoms exhibits a gradual increase followed by equilibration. At lower displacement cascades (0.01–0.10 keV), the vacancy-interstitial atoms predominantly cluster around CRPs, forming (Cv, Ci) shells. However, at higher displacement cascades (0.50–5.00 keV), the number of vacancy-interstitial atoms initially peaks, then declines, and eventually rises again, indicating that collision with CRPs generates a significant population of vacancy-interstitial pairs. These pairs tend to cluster around the periphery of CRPs, forming shells of vacancy-interstitial atoms. Moreover, as the displacement cascades increase, vacancy-interstitial atoms gradually appear within the interior of CRPs. These findings align with the outcomes obtained from phase-field simulation.

## Discussion

**Figure 6:**
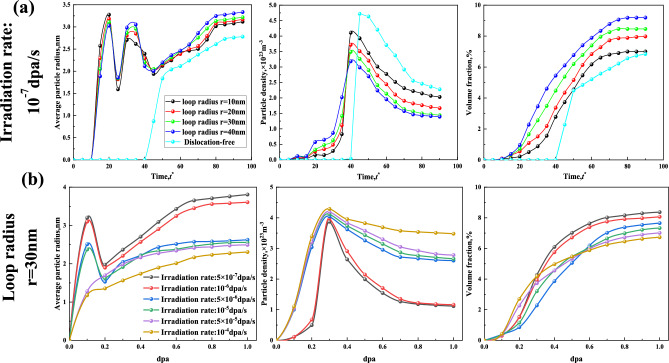
Plots showing the evolution of average particle radius, volume fraction, and particle density of CRPs under the different dislocation loop and different irradiation rates.

Figure 6a shows that the absence of dislocation loops leads to a rapid increase in average particle radius, volume fraction, and particle density, indicating the prompt formation of CRPs induced by irradiation. Subsequently, the average particle radius and volume fraction exhibit slow increments, while the particle density gradually decreases, eventually reaching equilibrium. The presence of dislocation loop reduces the gestation period for the nucleation of CRPs. The average particle radius increases rapidly, the volume fraction experiences a slower increase. This suggests that the formation of precipitates in the early aging stage is mainly influenced by the dislocation loop. Subsequently, the average particle radius first decreased and then increased, and the particle density continued to slowly increase, indicating that new dislocations assisted the nucleation and growth of precipitation. As aging progresses, the average particle radius first decreases and gradually increases, and the particle density first rapidly increases to the peak and then gradually decreases. This gradual process implies the formation and growth of radiation-induced CRPs.

The results in Fig. 6b suggest that the average particle radius, particle density, and volume fraction under irradiation rates ranging from 5 × 10^−7^ to 10^−6^ dpa/s are essentially similar to those under the influence of irradiation rate of 10^−7^ dpa/s and dislocation loops (r = 30 nm) as depicted in Fig. 6a. When the irradiation doses are below 0.2 dpa for irradiation rates of $5\times {10}^{-6}$ to ${10}^{-5}\text{ dpa}/\text{s}$, the average particle radius, particle density, and volume fraction are similar to those depicted in Fig. 6. However, as the irradiation dose exceeds 0.2 dpa, the average particle radius and volume fraction of CRPs progressively increase and approach equilibrium, while the particle density gradually decreases and also reaches equilibrium. This indicated that no new dislocations were generated around the irradiation-induced CRPs. Following the precipitation facilitated by dislocation loops, irradiation promotes the formation and growth of precipitates. At irradiation rates ranging from $5\times {10}^{-5}$ to ${10}^{-4}\text{ dpa}/\text{s}$, the average particle radius, particle density, and volume fraction initially experience a rapid increase, which then slows down before finally reaching a stable state. This observation indicates that irradiation consistently stimulates the formation and growth of CRPs throughout the entire process.

Chemical strengthening $\Delta {\sigma }_{chemical}$, coherence strengthening $\Delta {\sigma }_{coherency}$, modulus strengthening $\Delta {\sigma }_{modulus}$ and Orowan strengthening $\Delta {\sigma }_{\text{Orowan}}$ can be expressed as^55^:

11 $$\begin{aligned} \Delta \sigma_{chemical} = & \frac{2M}{{bLT\prime^{\frac{1}{2}} }} \cdot \left( {f^{in} {\varvec{b}}} \right)^{\frac{3}{2}} \\ \Delta \sigma_{coherency} = & \,4.1MG\varepsilon^{\frac{3}{2}} f_{CRPs}^{\frac{1}{2}} \left( {\frac{{R_{CRPs} }}{{\varvec{b}}}} \right)^{\frac{1}{2}} \\ \Delta \sigma_{modulus} = & \,M\frac{{G{\varvec{b}}}}{L}\left[ {1 - \left( {\frac{{E_{p} }}{{E_{m} }}} \right)^{2} } \right]^{\frac{3}{4}} \\ \Delta \sigma_{{{\text{Orowan}}}} = & \,0.84\left( {\frac{{2T^{\prime}}}{{R_{\alpha \prime } {\varvec{b}} \cdot \sqrt {\frac{2\pi }{{3f_{CRPs} }}} }}} \right) \\ \end{aligned}$$

where $M=3.06$ is Taylor factor. b is the Burgers vector. $T^\prime = G{\varvec{b}}^{2} /2$ is the line tension of the dislocation. ${f}^{in}$ is the interface energy. $L=0.866/{\left({R}_{CRPs}{N}_{CRPs}\right)}^\frac{1}{2}$ (${R}_{CRPs}$ and ${N}_{CRPs}$ are the radius and number density of CRPs, respectively) is the equivalent spacing of the precipitates in the direction of dislocation slip. $G=83GPa$ is the shear modulus of α-Fe (matrix). ε $\approx 1.5\left|\delta \right|$ is the coherent dependent variable, and $\delta$ is the mismatch degree between the precipitate and the matrix. ${f}_{CRPs}$ is the volume fraction of $CRPs$. ${E}_{p}$ is the line energy of dislocations in $CRPs$. ${E}_{m}$ is the line energy of dislocations in the matrix, and ${E}_{p}/{E}_{m}$ is assumed to be 0.987.

**Figure 7:**
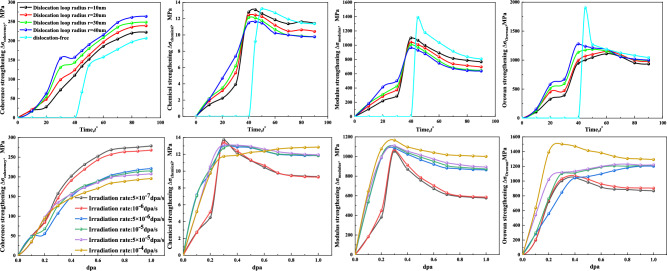
Strengthening contributions of $\Delta {\sigma }_{chemical}$, $\Delta {\sigma }_{coherency}$, $\Delta {\sigma }_{modulus}$ and $\Delta {\sigma }_{\text{Orowan}}$ under the synergistic effect of different dislocation loop and neutron irradiation.

The stress differences ($\Delta \sigma$) in coherency, chemical, modulus, and Orowan effects exhibit different patterns during the formation and growth of irradiation-induced CRPs, as shown in Fig. 7. $\Delta {\sigma }_{coherency}$ shows an initial rapid increase, followed by a gradual equilibrium. On the other hand, $\Delta {\sigma }_{chemical}$, $\Delta {\sigma }_{modulus}$, and $\Delta {\sigma }_{\text{Orowan}}$ increase rapidly to a peak, then decrease rapidly and eventually reach equilibrium. Among them, $\Delta {\sigma }_{modulus}$ and $\Delta {\sigma }_{\text{Orowan}}$ play a dominant role in reinforcing the material during the formation and growth of CRPs. Compared to the presence of dislocation loops, irradiation-induced CRPs result in a maximum value of $\Delta {\sigma }_{modulus}$, $\Delta {\sigma }_{\text{Orowan}}$, and total intensity at equilibrium. Furthermore, the radius of the dislocation loop affects the stress differences. $\Delta {\sigma }_{coherency}$ and $\Delta {\sigma }_{\text{Orowan}}$ increase with an increasing loop radius, while $\Delta {\sigma }_{chemical}$, $\Delta {\sigma }_{modulus}$, and total strengthening decrease with an increasing loop radius. The rate of irradiation also influences the stress differences. $\Delta {\sigma }_{coherency}$ is more prominent at lower irradiation rates. The peak of $\Delta {\sigma }_{chemical}$ is highest, and the equilibrium intensity is lowest at higher irradiation rates. The peak values of $\Delta {\sigma }_{modulus}$ and $\Delta {\sigma }_{\text{Orowan}}$ increase with increasing irradiation rate. In summary, $\Delta {\sigma }_{chemical}$, $\Delta {\sigma }_{coherency}$, $\Delta {\sigma }_{modulus}$ and $\Delta {\sigma }_{\text{Orowan}}$ exhibit distinct behaviors during the formation and growth of CRPs, with $\Delta {\sigma }_{modulus}$ and $\Delta {\sigma }_{\text{Orowan}}$ playing prominent reinforcing roles. The effects of dislocation loop radius and irradiation rate further influence the stress differences and overall strengthening.

Precipitation hardening ${\sigma }_{SC}$^56^ caused by CRPs can be expressed as

12 $$\begin{array}{c}{\sigma }_{SC}=\frac{\alpha MGb}{L}==0.8MGb\frac{\sqrt{{f}_{CRPs}}}{1.77r}\sqrt{1-{\left(\frac{{E}_{1}^{\infty }\text{log}\frac{r}{{r}_{0}}}{{E}_{2}^{\infty }\text{log}\frac{R}{{r}_{0}}}+\frac{\text{log}\frac{R}{r}}{\text{log}\frac{R}{{r}_{0}}}\right)}^{2}}\end{array}$$

where $M$ is the Taylor coefficient. $G=83\text{GPa}$ is the shear modulus of the matrix. $b$ is the size of the Burgers vector of the dislocation. $L=1.77r/{f}_{CRPs}^{1/2}$ is the average spacing of CRPs on (001) slip plane. $r$ is the radius and ${f}_{CRPs}$ is the volume fraction. ${E}_{1}^{\infty }$ and ${E}_{2}^{\infty }$ refer to the energy per unit length of dislocations in CRPs and matrix, respectively. ${E}_{1}^{\infty }/{E}_{2}^{\infty }$ equals ${G}_{1}/G=0.6$. ${G}_{1}$ is the precipitates shear modulus. ${r}_{0}=2.5b$ is the internal cutting radius, and $R=2500b$ is the external cutting radius^57^.

**Figure 8:**
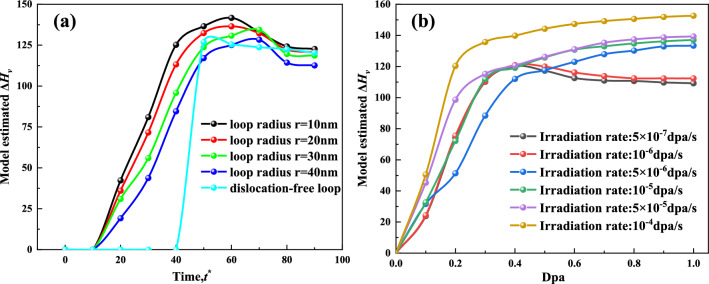
Hardening of CPRs with time in Fe–Cu–Mn–Ni–Al Alloys under synergistic-competitive effect of dislocation loop and irradiation.

In the presence of the synergistic-competitive effect of dislocation loops and irradiation, the hardness of irradiation-induced precipitates follows a specific pattern. This is depicted in Eq. (12) and illustrated in Fig. 8. At a low irradiation rate of (${10}^{-7}$ $\text{dpa}/\text{s}$), the hardness increases rapidly and reaches its peak quickly in the absence of dislocations. The presence of dislocation loop-assisted precipitates leads to a sharp increase in hardness, followed by a slower increase due to new dislocation-assisted precipitates. Finally, the hardness experiences another rapid increase due to irradiation-induced precipitates, reaching its peak. Subsequently, the hardness gradually decreases and approaches equilibrium. As the irradiation rate increases to ($5\times {10}^{-7}$ to ${10}^{-6}\text{ dpa}/\text{s}$), the hardness initially increases rapidly, reaches its peak, and then decreases slowly. The trend towards equilibrium becomes more pronounced. Interestingly, at higher irradiation rates, ranging from $5\times {10}^{-6}$ to ${10}^{-4}\text{ dpa}/\text{s}$, the hardness continues to increase without displaying a peak. The increase is rapid initially and then slows down, but there is no subsequent decrease in hardness. In summary, the hardness of irradiation-induced precipitates is influenced by the synergistic-competitive effect of dislocation loops and irradiation rate. The presence of dislocations, the irradiation rate, and the interactions between these factors determine the pattern of hardness evolution, including the speed of increase, the presence or absence of a peak, and the tendency toward equilibrium.

## Conclusion

The paper presents a phase-field model for a Fe–Cu–Mn–Ni–Al alloy, considering the interactions of cascade mixing, vacancy-interstitial atoms, and dislocation elastic fields. The research findings reveal that at low irradiation rates (${10}^{-7}$ to ${10}^{-6}\text{ dpa}/\text{s}$), dislocation loops play a crucial role in facilitating the formation of precipitates and generating new dislocations. As the irradiation proceeds, a significant amount of precipitation occurs. Vacancy-interstitial atoms tend to aggregate around dislocations and in the Cu-rich precipitates. The Cu-rich precipitates demonstrate a double-core-shell structure of Cu/(Cv, Ci)/(Mn, Ni, Al), where vacancy-interstitial atoms collect in the inner shell and (Mn, Ni, Al) cluster in the outer shell. Molecular dynamics simulations support the distribution of Mn, Ni, and vacancy-interstitial atoms during the displacement cascade, aligning with the phase-field model's outcomes. No new dislocations are created in the vicinity of the dislocation loop with increasing irradiation rates ($5\times {10}^{-6}$ to ${10}^{-5}\text{ dpa}/\text{s}$). Only the irradiation induces the formation of a large number of precipitates with the irradiation rate continues to increase ($5\times {10}^{-5}$ to ${10}^{-4}\text{ dpa}/\text{s}$). However, the paper does not account for the influence of grain boundaries. Therefore, it is suggested that further research investigates the interplay among dislocations, irradiation, grain boundaries, and precipitates in this model to better match the real-world conditions of Fe–Cu–Mn–Ni–Al multicomponent alloys.

### Supplementary Information

Supplementary Information.

## Data Availability

The datasets generated during and/or analysed during the current study are available from the corresponding author on reasonable request.
