# Supplementary material for: Dislocation loop and irradiation-induced synergistic-competitive mechanism in Cu-rich precipitates: a phase-field study
Source: Sci Rep. 2024 Jun 4;14:12767. doi: 10.1038/s41598-024-63632-5 (PMC11150390; doi:10.1038/s41598-024-63632-5)
Supplement: Supplementary file 1 — Supplementary Information. [file 41598_2024_63632_MOESM1_ESM.pdf]

Dislocation loop and irradiation-induced synergistic-competitive mechanism in Cu-rich precipitates: a phase-field study

Wenkui Yang1, Qingwei Guo1, Kaile Wang1, Pengya Lei1, Hua Hou1,3, Yuhong Zhao1,2,4,*

[1] School of Materials Science and Engineering, Collaborative Innovation Center of Ministry of Education and Shanxi Province for High-performance Al/Mg Alloy Materials, North University of China, Taiyuan 030051, P R China

[2] Beijing Advanced Innovation Center for Materials Genome Engineering, University of Science and Technology Beijing, Beijing 100083, P R China

[3] A School of Materials Science and Engineering, Taiyuan University of Science and Technology, Taiyuan 030024, China

[4] Institute of Materials Intelligent Technology, Liaoning Academy of Materials, Shenyang 110004, China

* Corresponding authors: Yuhong Zhao (zhaoyuhong@nuc.edu.cn).

Supplementary information

Appendix A

The free energy chemical free energy density f_{ch} can be expressed as

$$f_{ch} = E_V^f c_V + E_I^f c_I + \sum_i G_i^0 c_i + RT \sum_i \begin{pmatrix} c_i ln c_i + c_V ln(c_V) \\ + c_I ln(c_I) \end{pmatrix} + \sum_i \sum_{j>i} L_{i,j} c_i c_j + \sum_i \sum_{j>i} \sum_{k>j} L_{i,j,k} c_i c_j c_k \qquad (A.1)$$

where $E_V^f = 1.12\text{eV}$ and $E_I^f = 1.48\text{eV}$ are the formation energies of vacancies and interstitial atoms. G_i^0 is the Gibbs free energy of the pure component i. $L_{i,j}$ and $L_{i,j,k}$ are the binary and ternary interaction parameters, respectively. The nterfacial energy density f_{in} can be written as

$$f_{in} = \frac{1}{2} \sum k_{c_i} (\nabla c_i)^2 + \frac{1}{2} k_V (\nabla c_V)^2 + \frac{1}{2} k_I (\nabla c_V)^2 \qquad (A.2)$$

where k_{c_i}, k_V, and k_I are the gradient energy coefficient of element i, vacancies and interstitial atoms.

Gibbs free energies of pure i elements (G_i^a) and the binary $L_{i,j}$ and ternary interaction parameters:

$G_{Fe}^a = 0$

$G_{Cu}^a = 4017 - 1.255T$

$$G_{Mn}^{a} = -3235.3 + 127.85T - 23.7\text{Tln }T - 0.0074271T^2 + 60000/T$$

$$G_{Ni}^{a} = 8715.084 - 3.556T$$

$$G_{Al}^{a} = -1193.24 + 218.235446T - 38.5844296T\ln T + 18.5319823\text{E} - 3T^2 - 5.764227\text{E} - 6T^3 + 74092T^{-1}$$

$$L_{Fe,Cu} = 41033 - 6.022T$$

$$L_{Fe,Mn} = -2759 + 1.237T$$

$$L_{Fe,Ni} = -956.63 - 1.28726T + (1789.03 - 1.92912T)(c_1 - c_4)$$

$$L_{Fe,Al} = -122960 + 31.989T - (2945.2)(c_1 - c_5)$$

$$L_{Cu,Mn} = 11190 - 6T - 9865(c_2 - c_3)$$

$$L_{Cu,Ni} = 8366 + 2.8T$$

$$L_{Cu,Al} = -104600 + 27.5T - (9800 + 20T)(c_2 - c_5)$$

$$L_{Mn,Ni} = -51638.31 + 3.64T + 6276(c_3 - c_4)$$

$$L_{Mn,Al} = -120077 + 52.851T - (-40652 + 29.276T)(c_3 - c_5)$$

$$L_{Ni,Al} = -264500 - 119T + 23T\ln T - (-107000 + 559T - 67T\ln T)(c_4 - c_5) + (414000 - 800T + 95T\ln T)(c_4 - c_5)^2 - (-118000 + 970T - 99T\ln T)(c_4 - c_5)^3$$

$$L_{Fe,Cu,Mn} = 30000$$

$$L_{Fe,Cu,Al} = -15000c_1 + 35000c_2 - 160000c_5$$

$$L_{Fe,Cu,Ni} = L_{2,3,4} = L_{1,3,4} = 0$$

$$L_{Fe,Mn.Al} = 4184$$

$$L_{Fe,Ni,Al} = (-27000 + 1.1T)c_1 + (-204500 + 107T)c_4 + (-110700 + 34T)c_5$$

$$L_{Cu,Mn,Al} = 36000$$

$$L_{Cu,Ni,Al} = (-28000 + 40T)c_4 + (-133000 + 40T)c_2 + (-200000 - 100T)c_5$$

$$L_{Mn,Ni,Al} = (45897.48 - 81.07T)c_3 + (110124.13 - 163.65T)c_4 + (187015.11 - 106.26T)c_5$$

The lattice expansion coefficients caused by the introduction of Cu, Mn, Ni, Al atoms into the Fe matrix are $\varepsilon_2^0 = 3.29 \times 10^{-2}$, $\varepsilon_3^0 = 5.22 \times 10^{-4}$, $\varepsilon_4^0 = 4.75 \times 10^{-4}$, $\varepsilon_4^0 = 4.75 \times 10^{-4}$, respectively.

The elastic constants of matrix and precipitation selected in the simulation are as follows: $C_{11}^m = 228GPa$, $C_{12}^m = 132GPa$, $C_{44}^m = 116.5GPa$, $C_{11}^p = 169GPa$, $C_{12}^p = 122GPa$, $C_{44}^p = 75.3GPa$.

The atomic mobility of M_i^{th} can be expressed as

$$M_i^{th} = \frac{D_i^{th}}{RT} \tag{A.3}$$

where R is the gas constant ($R = 8.314472J/(mol \cdot K)$) and $T = 773K$ is the temperature. The atomic diffusion coefficient D_i can be written as

$$D_i^{th} = D_i^0 exp\left(\frac{-Q_i}{RT}\right) \quad (A.4)$$

Where Q_i is the diffusion activation energy of i atom

Table A.1 The basic dynamic parameters of alloying elements

element	Frequency factor D_i^0 (m^2/s)	Diffusion activation energy Q_i (J/mol)	Diffusion coefficient D_i
Fe	1.00×10^{-4}	2.94×10^5	1.36×10^{-24}
Cu	4.70×10^{-5}	2.44×10^5	1.53×10^{-20}
Mn	1.49×10^{-5}	2.33×10^5	3.02×10^{-21}
Ni	1.40×10^{-5}	2.46×10^5	3.34×10^{-21}
Al	5.35×10^{-5}	2.71×10^5	3.61×10^{-22}

Appendix B

A short-range ordered model was obtained by melting/quenching and hybrid MC/MD simulations of the optimized structure. Melting/quenching was obtained by successive heating, keeping high temperature, cooling and keeping low temperature on the basis of a stochastic solid solution model. The process was implemented in the NVT system.

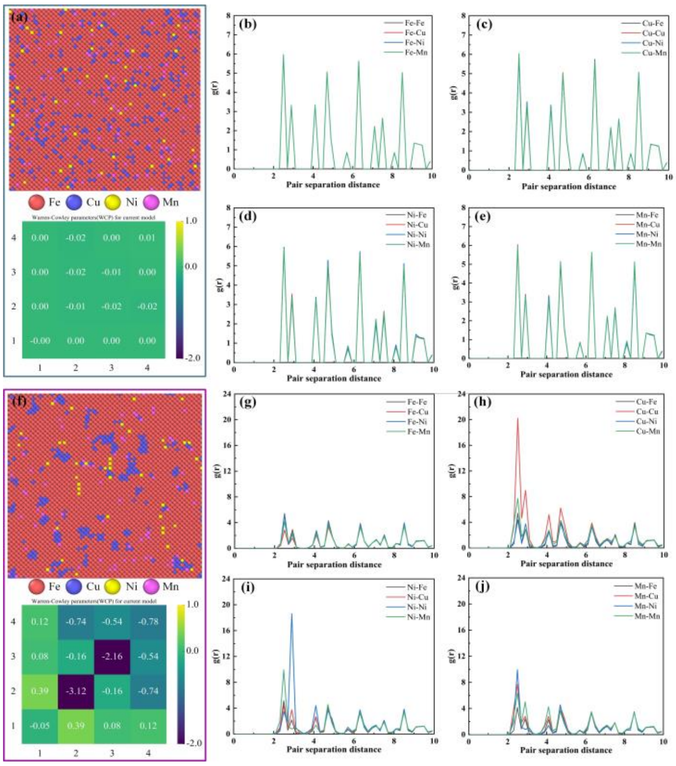

Fig. S1 (a) Stochastic solid solution model and Warren-Cowley parameters, (b-e) radial distribution functions of the stochastic solid solution model, (f) model and Warren-Cowley parameters after melting/quenching and hybrid MC/MD simulations, (g-j) radial distribution functions of the model after melting/quenching and hybrid MC/MD simulations

It can be found that the Warren-Cowley parameter in the stochastic solid solution model is close to 0, indicating that it is in a random atomic distribution, see Fig. S1 (a). In order to quantify the pairwise trend of Fe-Cu-Mn-Ni alloys, we calculated the radial distribution function (RDF), see Fig. S1 (b-e). We note that the radial distribution function curves between atom pairs in the stochastic solid solution model overlap, indicating a random distribution of its elements. After melting/quenching followed by hybrid MC/MD simulation, Cu elements undergo significant clustering, see Fig. S1 (f). The Warren-Cowley parameter shows that Cu-Cu and Ni-Ni can undergo significant clustering, and Cu-Mn and Ni-Mn have a tendency to cluster. The radial distribution function confirms the clustering of Cu and Ni elements. Based on the distribution of atoms, our EAM potential looks to successfully simulate the interaction and bias aggregation behavior between elements in Fe-Cu-Mn-Ni alloys. This result well validates the phase-field simulation results.

Appendix C

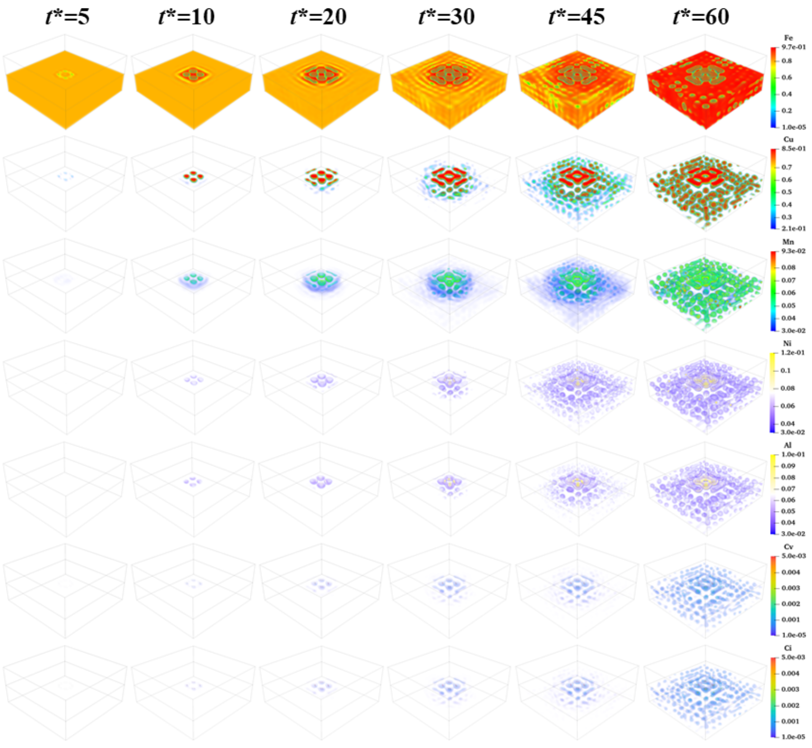

Fig. S2 3D atomic distribution results under the synergistic effect of dislocation loop (r=10nm) and neutron irradiation ($10^{-7}\ dpa/s$)

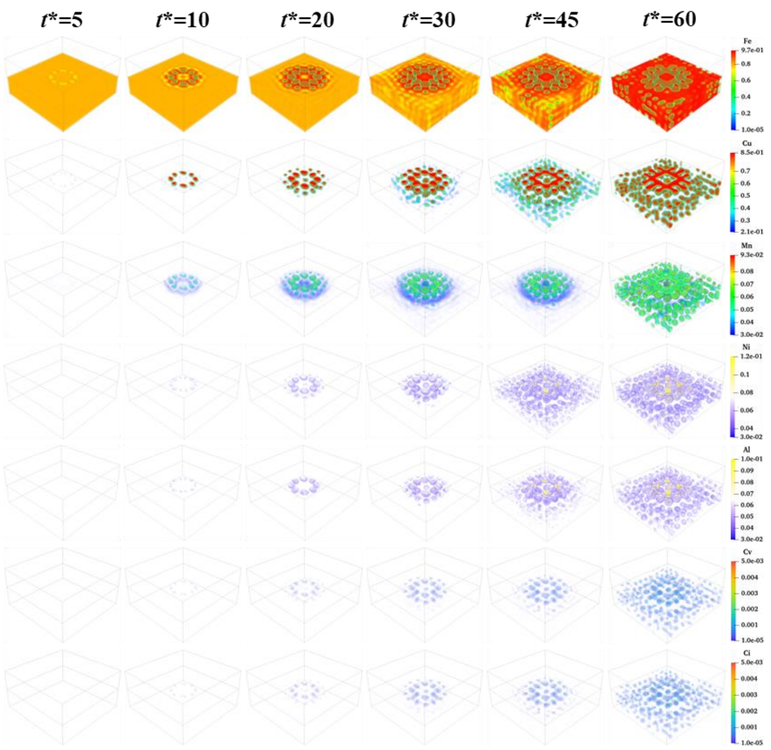

Fig. S3 3D atomic distribution results under the synergistic effect of dislocation loop (r=20nm) and neutron irradiation ($10^{-7}\ dpa/s$)

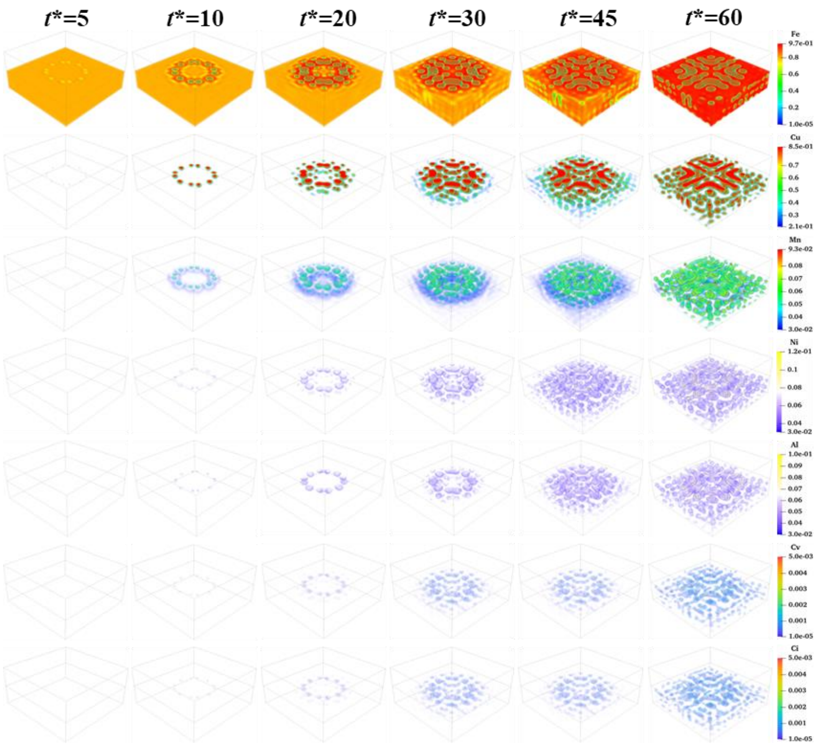

Fig. S4 3D atomic distribution results under the synergistic effect of dislocation loop (r=30nm) and neutron irradiation ($10^{-7}\ dpa/s$)

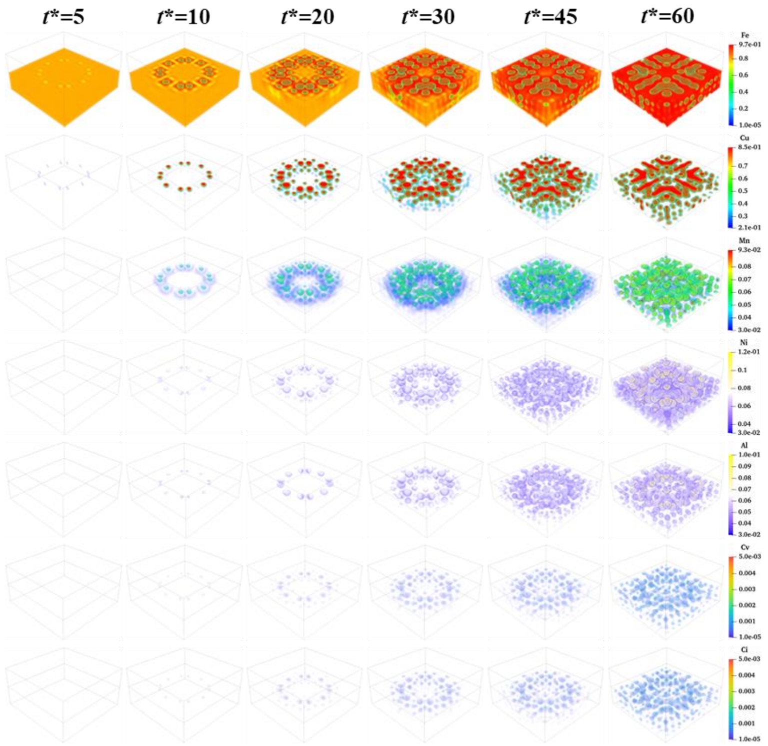

Fig. S5 3D atomic distribution results under the synergistic effect of dislocation loop (r=40nm) and neutron irradiation ($10^{-7}\ dpa/s$)

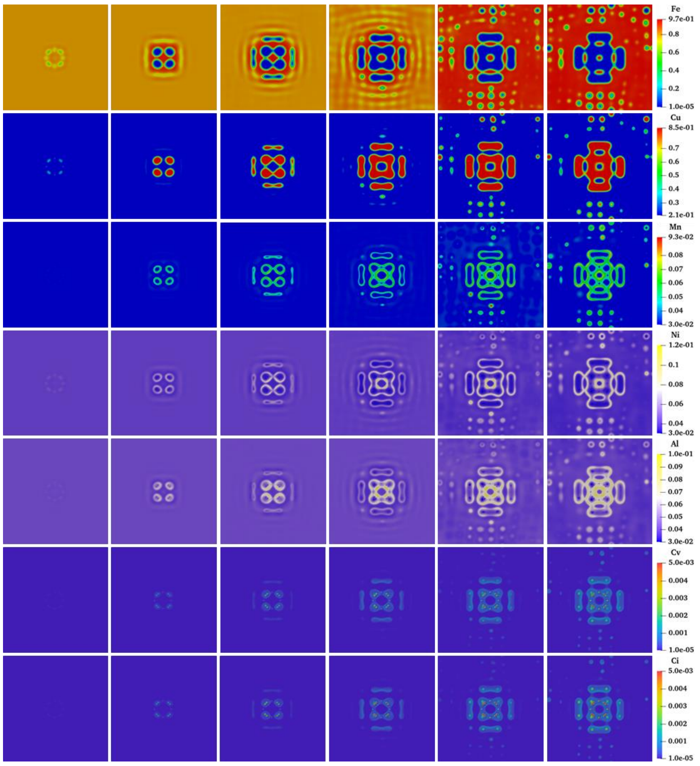

Fig. S6 Evolution of the element distribution in the (001) plane with time under the synergistic effect of dislocation loop (r=10nm) and neutron irradiation (10^{-7} dpa/s)

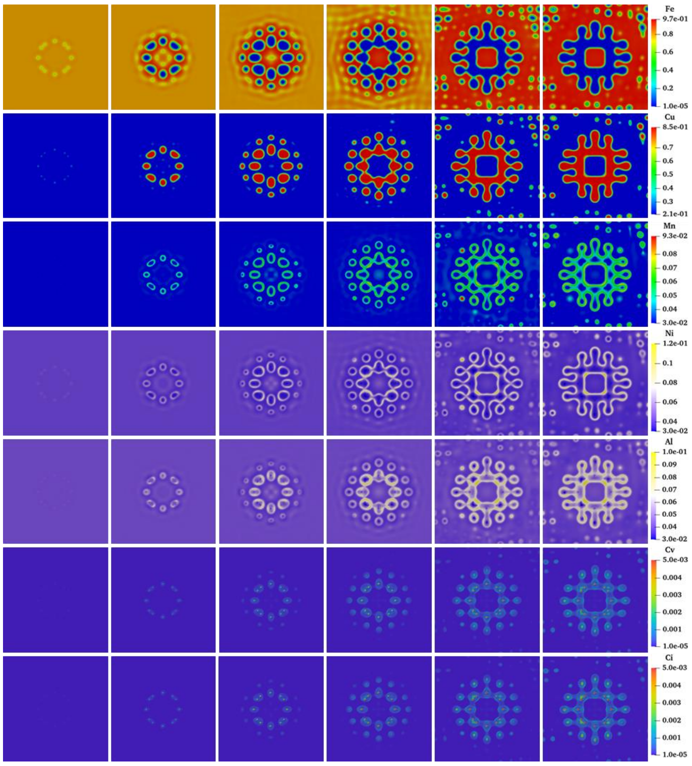

Fig. S7 Evolution of the element distribution in the (001) plane with time under the synergistic effect of dislocation loop (r=20nm) and neutron irradiation (10^{-7} dpa/s)

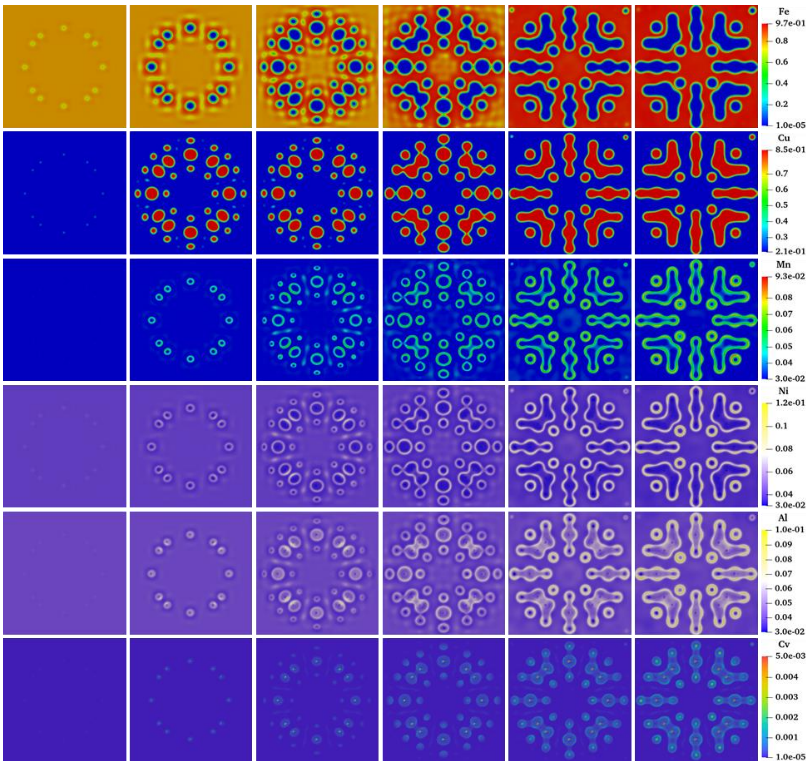

Fig. S8 Evolution of the element distribution in the (001) plane with time under the synergistic effect of dislocation loop (r=40nm) and neutron irradiation ($10^{-7}\ dpa/s$)
